# Functional trait diversity of Cyclanthaceae and its convergent evolution with Araceae in Neotropical forests

**DOI:** 10.7717/peerj.15557

**Published:** 2023-07-18

**Authors:** Erin C. Riordan, Orlando Vargas Ramirez, Philip W. Rundel

**Affiliations:** 1 Department of Ecology and Evolutionary Biology, University of California, Los Angeles, Los Angeles, California, United States; 2 La Selva Biological Station, Puerto Viejo de Sarapiqui, 41001, Costa Rica

**Keywords:** Growth form, Hemiepiphyte, Epiphyte, Heteroblasty, Araceae, Convergent evolution

## Abstract

The Cyclanthaceae comprise a relatively small family of about 230 species and 12 genera in the Pandanales that is widespread in wet Neotropical forests. The great majority of species can be divided into three growth forms (understory herbs, epiphytes, and root-climbing hemiepiphytes) that share functional traits with similar growth forms present in the Araceae, a member of the Alismatales and not closely related. Our objectives were first to characterize the diversity, functional growth forms, and ecological traits of Cyclanthaceae at the La Selva Biological Station. Specific functional leaf and canopy traits of terrestrial herbs and epiphytes are very similar and associated with ecological success in both families. We further examined the functional traits of root-climbing hemiepiphytes, a specialized growth form that links the two families but rare in other families and argue that their specialized functional traits allow them to be considered as a distinct functional growth form. A key trait in distinguishing hemiepiphytes which are rare outside of the Cyclanthaceae and Araceae is the severance of the main stem hydraulic connection to the soil early in plant development. We used field data to examine the possible evolutionary pathways of developmental and ecological transition from terrestrial to hemiepiphyte growth forms. The broader ecological success of hemiepiphytic Araceae compared to Cyclanthaceae is hypothesized to result from the presence of heteroblasty in developing stems and leaves which allows more efficient utilization of complex canopy light environments of wet tropical forests.

## Introduction

The Cyclanthaceae comprises a relatively small Neotropical family consisting of 12 genera and about 230 species. The family is notable for the abundance and ecological significance of many of its species in wet tropical forest habitats over its range of distribution from southern Mexico to Brazil and Bolivia. Cyclanthaceae have their highest diversity at both the generic and species level in the wet Choco and montane forests of the northern Andes of South America (Table 1). The early diversification of Cyclanthaceae has been estimated to date to the Paleocene-Eocene period in South America (Leal et al., 2022). The origin of most genera has been hypothesized to have occurred in the Paleocene of the Tumbes-Chocó-Magdalena region, suggesting an early origin for this biodiversity hotspot. The current distribution of Cyclanthaceae has been influenced by the uplift of the Andes and the opening of the South America dry diagonal (Leal et al., 2022).

**Table 1:** Species, biogeographic ranges, and dominant growth forms of genera of Cyclanthaceae.

Genus	Total	Costa Rica	OTS	Geographic range	Growth form
*Asplundia*	100	19	8	S Mexico to Brazil	Terrestrial, Hemiepiphyte
*Carludovica*	4	4	2	Mexico to N Andes	Terrestrial
*Chorigyne*	7	4	1	Costa Rica, Panama	Epiphyte
*Cyclanthus*	2	1	1	S Mexico to Bolivia	Terrestrial
*Dianthoveus*	1	0	0	N Andes	Terrestrial
*Dicranopygium*	54	6	2	S Mexico to Peru	Terrestrial, Rheophyte
*Evodianthus*	1	1	1	Costa Rica to N South America	Hemiepiphyte
*Ludovia*	3	1	1	Nicaragua to N South America	Epiphyte
*Schultesiophytum*	1	0	0	N Andes	Terrestrial
*Sphaeradenia*	53	12	2	Nicaragua to N South America	Epiphyte
*Stelestylis*	5	0	0	Venezuela, Guianas	Terrestrial
*Thoracocarpus*	1	1	0	Costa Rica to N South America	Hemiepiphyte
Total	232	49	18		

The great majority of Cyclanthaceae display distinctive growth forms that act as plant functional groups across generic lines. In multiple ways these diverse life forms parallel those that have been described for the larger Araceae (Croat, 1988; Croat & Ortiz, 2020). Despite the distant relationship of the two families, both are dominated by a mix of three functional growth forms—terrestrial herbs, epiphytes, and most notably root-climbing hemiepiphytes. This later growth form is rare in other Neotropical families and is characterized by distinctive functional traits. We refer to these as root-climbing hemiepiphytes, distinguished from what have been termed primary hemiepiphytes which begin their lives as true epiphytes (Putz & Holbrook, 1986; Holbrook & Putz, 1996).

The genus *Asplundia* forms the most diverse group of Cyclanthaceae with 100 species over its broad geographic distribution that matches that of the family. These species include a mixed array of terrestrial herbs and hemiepiphytes. Second in species richness is *Dicranopygium* with 54 described species. These species are primarily terrestrial herbs, with a few taxa that may become a low-growing root-climber as a hemiepiphyte. Also present are a number of herbaceous rheophyte species specialized or growing on rocks in fast-moving streams. The third large genus is *Sphaeradenia* with 53 species occurring typically as epiphytes.

For a variety of reasons, the Cyclanthaceae remains relatively poorly studied. Field collected specimens have often been misidentified as palms or aroids which they can resemble. Research on the family has been influenced by the limited amount of museum records available to aid in morphological and biogeographic studies. The large leaf size and thick stems of Cyclanthaceae which make them challenging to mount on standard herbarium sheets (Hammel, 1987). Neither the Arecaceae nor Araceae are closely related to the Cyclanthaceae which are placed in the Order Pandanales along with five smaller families (Angiosperm Phylogeny Group, 2016). The Cyclanthaceae is most closely related to the Pandanaceae which includes five genera and about 580 species in the Paleotropics from West Africa to the eastern Pacific.

Past studies of the Cyclanthaceae have centered on Costa Rica which is home to nine genera and 49 species in the family (Table 1). Much of this research has taken place at La Selva Biological Station in the northeastern Atlantic lowlands. The wet tropical forests of La Selva contain 18 species of Cyclanthaceae, with a good representation of genera and of plant functional groups typical of the family over its full range. By comparison, Barro Colorado Island in Panama has a flora of only five species of Cyclanthaceae with four of these terrestrial herbs (Croat, 1978).

**Figure 1:**
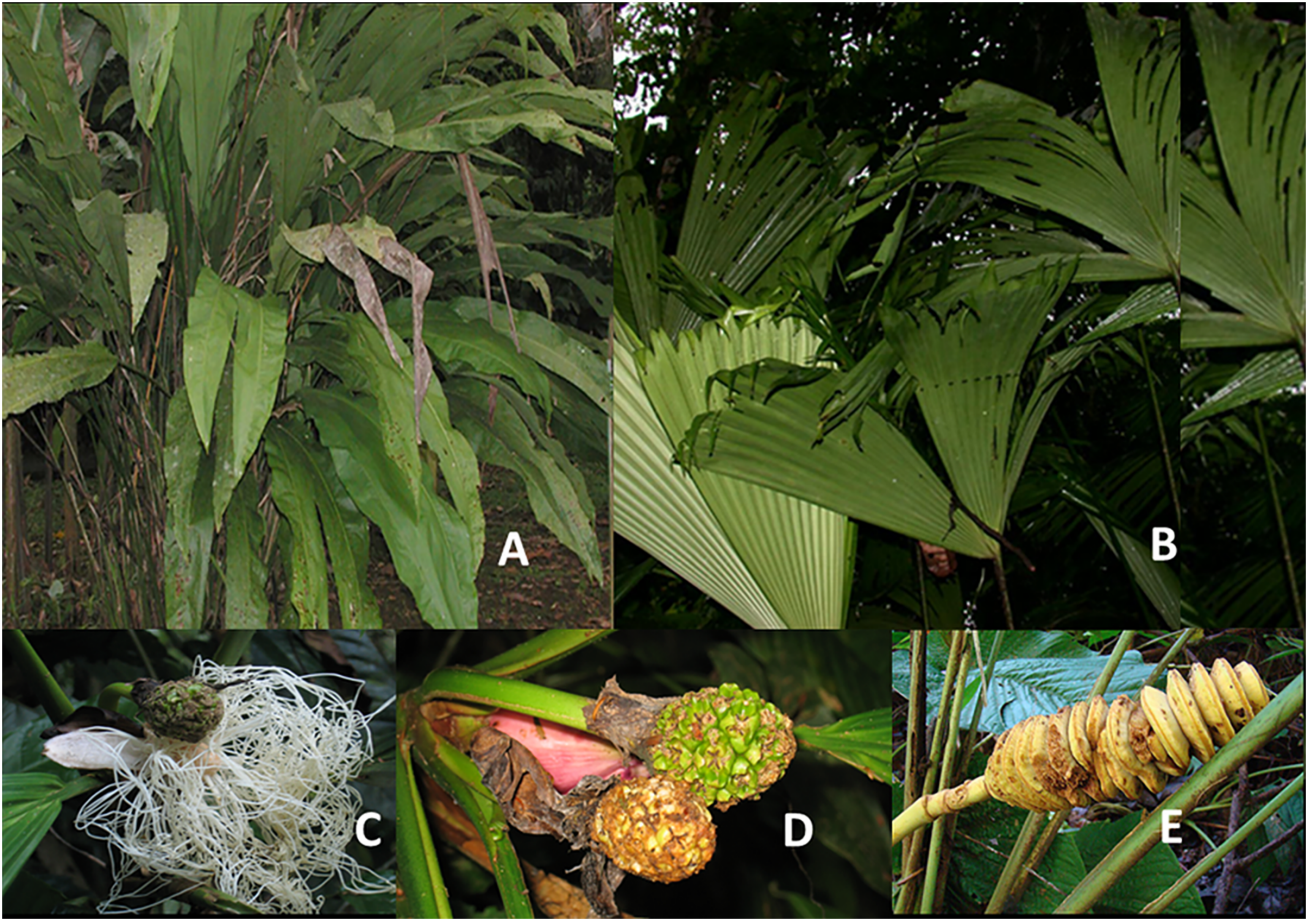
Leaf and reproductive structure of Cyclanthaceae. (A) *Cyclanthus bipartitus*. (B) *Carludovica sulcata*. (C) Monecious inflorescence of *Evodianthus funifer* with sterile staminoids which help attract beetle pollinators. (D) Female infructescence of *Evodianthus funifer* which is typical of the all genera except *Cyclanthus* (E) ring structure of female infructescence in *Cyclanthus bipartitus*.

Members of the Cyclanthaceae are monecious perennials with growth forms that include understory herbs, epiphytes, and most significantly root-climbing hemiepiphytes. With rare exceptions the leaves are bifid and petiolate, with 1–3 costa The family is divided into two subfamilies. The Cyclanthoideae includes the single genus *Cyclanthus* (Fig. 1A) and is characterized by densely crowded staminate and pistillate flowers arranged in alternate whorls. The Carludovicoideae, which includes all other genera (Fig. 1B), has a spirally arrangements of pistillate flowers surrounded by four staminate flowers. Pistillate flowers in most species have extended staminodes (Fig. 1C) which produce an odor attractant to pollinating beetles (Franz, 2007). Fruits in most species are either free or joined in a fleshy syncarp in most species (Fig. 1D), or in dried rings in *Cyclanthus* (Fig. 1E). Stems are typically lignified, vary from short to 10 m or more in length, and may be branched or unbranched.

Much of the research interest on the family has focused on its highly specialized pollination system relying on thermogenesis to attract a weevil pollinator (Beach, 1982; Eriksson, 1994; Franz, 2007; Teichert, Dötterl & Gottsberger, 2018). In addition to the plant functional traits of growth form, the Cyclanthaceae shares many functional traits of reproductive biology with the Araceae in pollination modes (Gottsberger, 1991; Silberbauer-Gottsberger et al., 2001) and seed dispersal (Cockle, 2001; Henry & Jouard, 2007).

Wet tropical rain forests with their species richness and diversity have been used for many years as case studies to compare leaf functional traits between tropical and temperate forest species. Functional traits in a complex light environment in tropical forests present an interesting example of the leaf economic spectra across a broad range of species and different climate regimes (Santiago & Wright, 2007; Sterck et al., 2011). More recently there have been examinations of plant functional traits in specific groups of tropical forest growth forms. These include quantification of leaf and canopy functional traits for broad-leaved understory herbs (Rundel et al., 2020), epiphytic aroids (Gerst et al., 2021), and epiphytes broadly (Wagner, Wanek & Zotz, 2021; Hietz et al., 2022).

Our objectives in this article have been first to characterize the diversity, functional growth forms, and ecological traits of Cyclanthaceae at the La Selva Biological Station. We compare and contrast these functional life form traits of Cyclanthaceae with those described for the Araceae (Croat, 1988; Croat & Ortiz, 2020). This large family has approximately 3,650 species and a worldwide biogeographic range with high species diversity and endemism in wet tropical forests. We compared the two families with respect to functional leaf and canopy traits of terrestrial herbs and epiphytes which are ecologically successful in both families. We further examined the characteristics root-climbing hemiepiphytes and argue that this growth form can be considered as a distinct functional group. We use field data to examine the possible evolutionary pathways of developmental and ecological transition from terrestrial to hemiepiphyte growth form. We consider how the differences in the lifeform spectra of the two families beyond major adaptive traits can help explain the differences in diversity and ecological success of these two families in a wet tropical rainforest.

## Materials and Methods

Hemiepiphyte diversity and distribution were studied in old-growth forests at La Selva Biological Station in the Caribbean lowlands of northeastern Costa Rica, Heredia Province (10°28′N, 83°59′W). The region is classified as tropical wet forest (Cooley, Reich & Rundel, 2004). The mean annual temperature is 26 °C, with little variation between months. Mean annual rainfall is about 4,600 mm, peaking in June through July and November through December (Graham et al., 2021). A drier period occurs in March, though monthly rainfall never falls below 100 mm during the dry season. Research permits were granted by the “Comisión Institucional de Biodiversidad” (Institutional Biodiversity Committee, University of Costa Rica; resolution VI-8315-2014) and authorized by La Selva Biological Station.

Using published morphological descriptions of the Costa Rican Cyclanthaceae and Araceae (Hammel, 2003; Grayum, 2003), as well as our collective field experience, we divided the species from each family into functional morphologies—understory herb (terrestrial, epilithic, rheophyte), epiphyte, and root-climbing hemiepiphyte (Hammel, 1986; Hammel, 2003; Grayum, 2003). Data on leaf functional traits of understory herbs in these families were extracted in the same manner as in published surveys of these traits (Rundel et al., 2020). Traits analyzed were leaf area, leaf specific area, leaf nitrogen content, leaf specific nitrogen content, leaf δ^13^C, and leaf water content. Mean values were based on samples five individual each of five species of Cyclanthaceae (*Asplundia uncinata*, *A. sleeperae*, *Cyclanthus bipartitus*, *Carludovicaulcatea*, and *Dicranopygium wedelii*) and nine species of Araceae (*Anthurium ochranthum*, *Dieffenbachia longispatha*, *D. toduzii*, *Philodendron grandipes*, *Spathiphyllum friedrichsthallii*, *S. fulvovirens*, *S. laeve*, *S. phrynifolium*, and *S. wendlandii*). Multiple voucher specimens for each study species are archived in the herbarium of the La Selva Biological Station (LSCR). This collection was founded in 1995 and contains more than 10,000 pressed plant specimens, collection numbers are available in Supplemental File 1.

We carried out field measurements of the presence or absence of a severed main stem connection to ground in young plants of five species of hemiepiphytic cyclanths and six species of hemiepiphytic aroids to evaluate the longevity of the main stem connection to the sample sizes were 20–30 individuals of each species. The height of the stem break above ground-level and the maximum height of the stem above ground level were measured.

## Results

### Growth form diversity of cyclanthaceae

The 49 Costa Rican species of Cyclanthaceae were divided into three functional growth form—understory herbs (terrestrial, epilithic, and rheophytic), epiphytes, and root-climbing hemiepiphytes.

### Understory herbs

**Figure 2:**
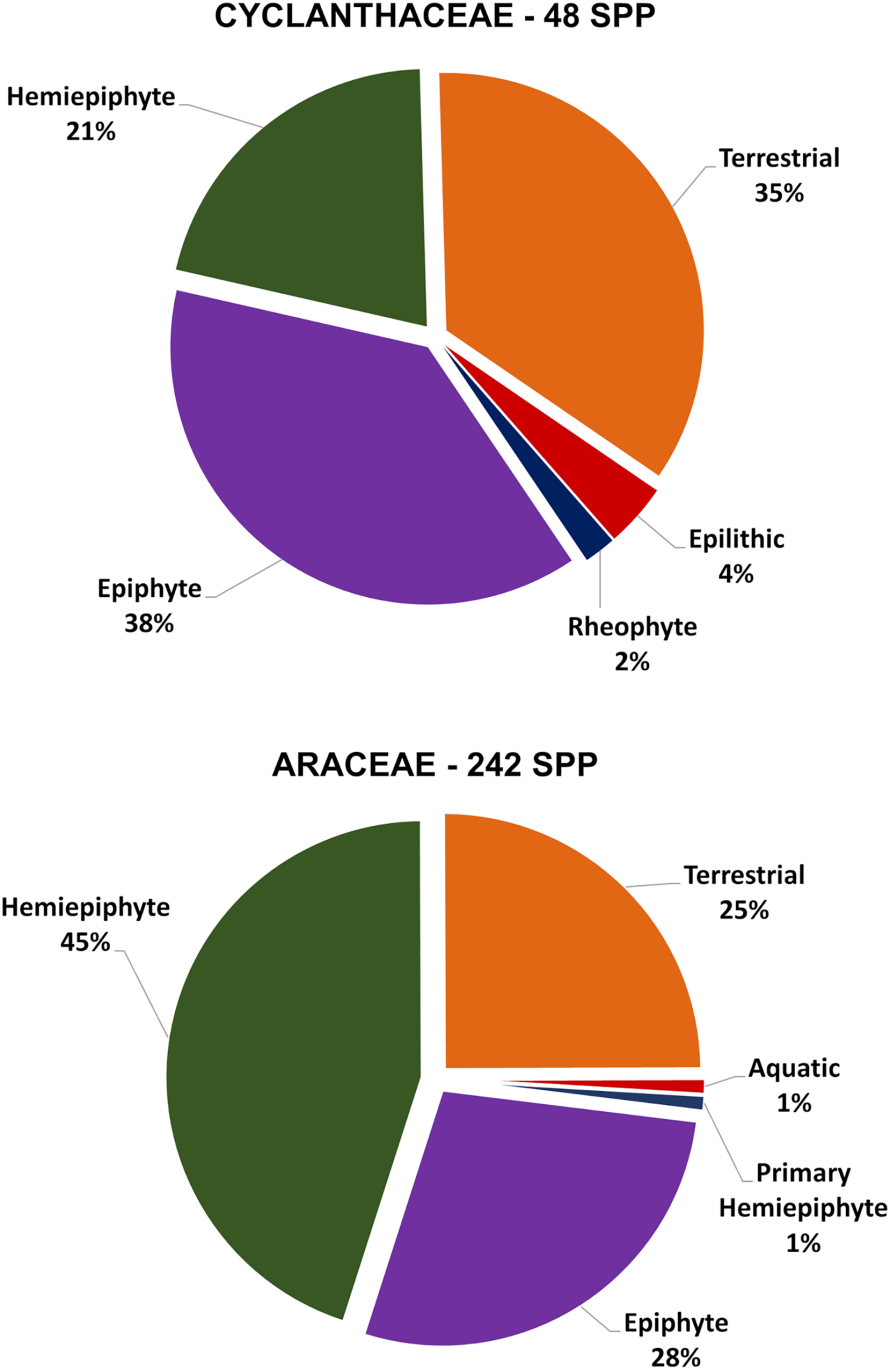
Comparative relative distribution of functional plant growth forms within the Costa Rican flora of Cyclanthaceae and Araceae.

**Figure 3:**
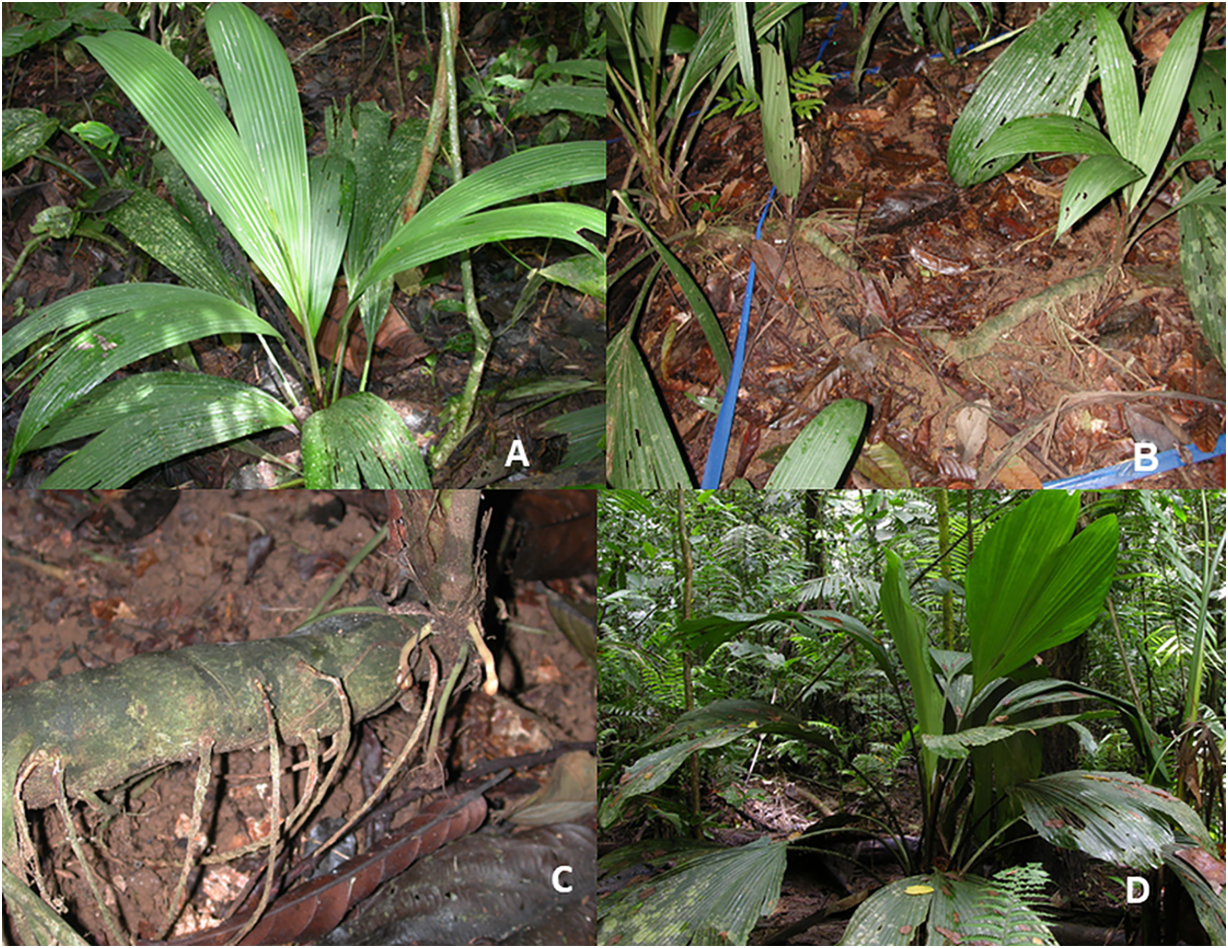
Understory Cyclanthaceae herbs. (A) *Asplundia uncinata*. (B) Connecting rhizomes of *A. uncinata*. (C) Rhizome of *A*. *uncinata*. (D) *Asplundia sleeperae*. Photos by Jennifer Sun.

Using data for all of Costa Rica, understory and other terrestrial herb species comprise 35% (17 species) of the Cyclanthaceae (Fig. 2). A key species is *Asplundia uncinata* (Fig. 3A) which forms a greater area of ground cover and biomass than any other broadleaf herb species in the shaded understory of the primary forest at La Selva (Rundel et al., 2020). This species of understory herb grows to 1–2 m in height with an extensive system of thick underground rhizomes that produce clonal stands that may extend for 10 m or more (Figs. 3B and 3C).

Although typically terrestrial in growth habit, *A. uncinata* can occasionally be observed using adventitious anchor roots to climb a short distance up a host tree. Individuals with attachments for root-climbing generally extend upward no more than 1–2 m above the ground and commonly lose their primary stem connection to the soil (Table 2). With development, the primary stem is severed, and small secondary roots slowly develop new connections to the soil. This type of flexibility in rooting behavior may be a transitional root-climbing life history, as discussed below.

**Table 2:** Comparative traits of secondary hemiepiphytic Cyclanthaceae and Araceae in natural populations at the La Selva Biological Station. Field measurements on young plants for mean heights of growth and height of point of stem severance, and frequency.

Species	*n*	Stem height (m)	Height of stem severance (m)	Frequency of stem severance (%)
**Cyclanthaceae**				
*Asplundia multistaminata*	10	2.5	1.0	100
*Asplundia uncinata*	20	1.1	0.4	85
*Asplundia utilis*	20	1.9	0.8	100
*Dicranopygium umbrophilum*	20	0.9	0.7	100
*Evodianthus funifer*	20	2.0	1.1	100
**Araceae**				
*Anthurium pentaphyllum*	20	1.0	1.8	55
*Anthurium subsignatum*	20	1.1	2.7	85
*Philodendron alliodorum*	20	1.4	1.1	75
*Philodendron inaequalaterum*	15	1.0	1.0	67

A second understory species of this genus, *Asplundia sleeperae* (Fig. 3D), displays very different traits. It is a robust terrestrial herb 1–2 m in height and relatively rare. It favors shaded understory habitats of primary forest on alluvial soils and is absent from swampy areas and secondary forests. Unlike *A. uncinata*, we have not observed any case of vegetative reproduction from underground rhizomes in *A. sleeperae*, and it has never been observed to climb.

**Figure 4:**
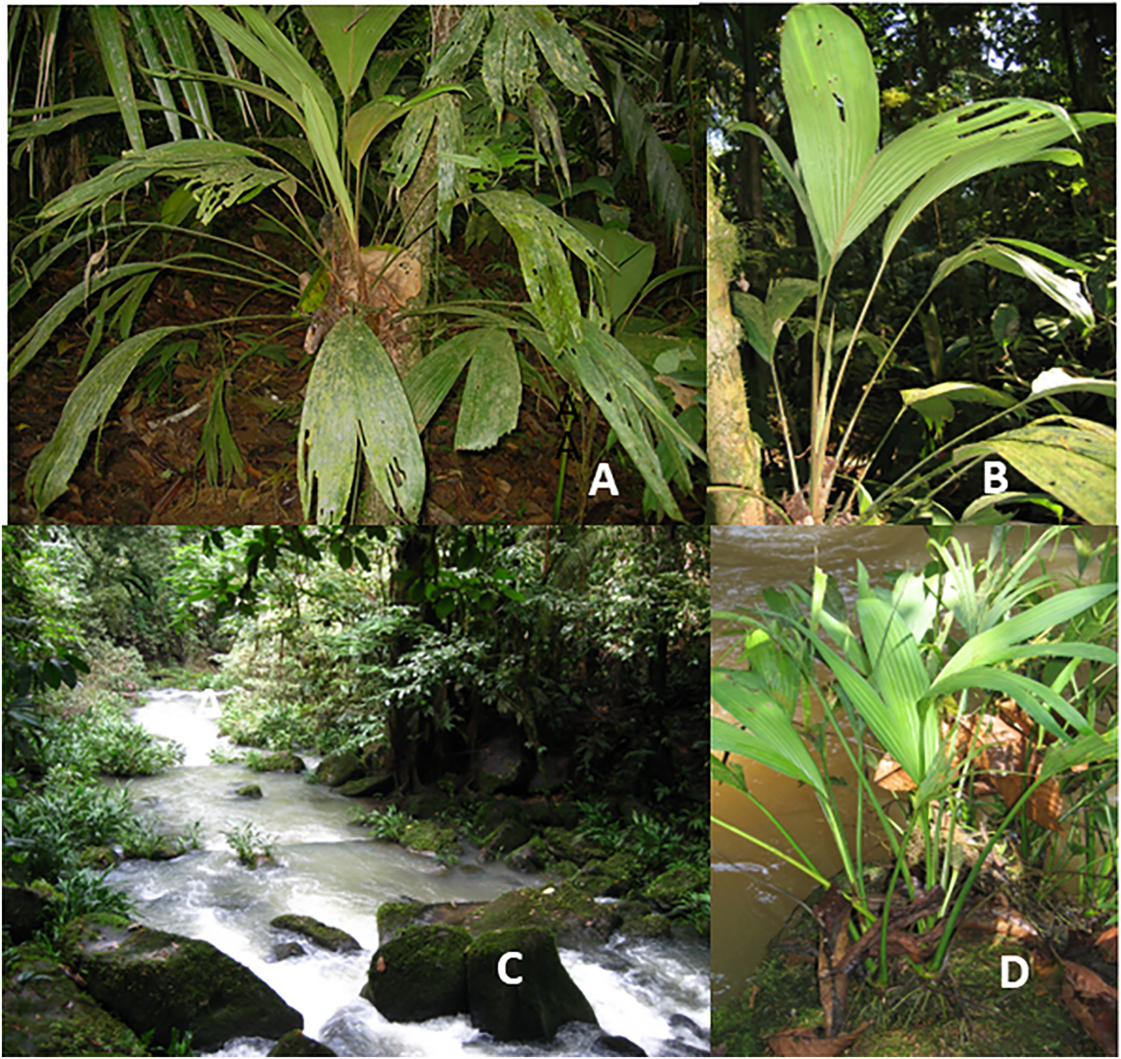
Epilithic and rheophytic Cyclanthaceae. (A) *Dicranopygium umbrophilum* with remaining soil attachment. (B) *D. umbrophilum* with trunk attachment. (C) Rheophytic habitat of *Dicranopygium wedelii*. (D) *D. wedelii*.

*Dicranopygium umbrophilum* (Fig. 4A) is a relatively small understory herb, usually no more than 0.5–1 m in height. Although present in low frequencies on well-drained residual soil, it becomes a dominant understory herb on humid swampy and lowland alluvial soils with a high soil moisture content and humid air in the understory at La Selva. Under these swampy conditions, individual plants typically attach themselves to stems or trunks of trees, changing their growth form to that of root-climbing hemiepiphytes. However, they limit their climb to no more than 1–2 m above the soil level. At this stage all primary stem connection to the soil is lost and the plants begin to develop secondary soil linkage with aerial roots (Table 2). As with occasional root-climbing seen in *Asplundia uncinata*, the common alteration of growth form to root-climbing in *D. umbrophilum* suggests an intermediate stage of adaptive change in moving from well drained terrestrial soils to water saturated swamp soils.

*Cyclanthus bipartitus* (Fig. 1A) is a tall acaulescent understory herb reaching up to 3 m in height. Its mature leaf blades are deeply divided almost to the base. A large underground rhizome allows for vegetative reproduction. Although highly variable in morphology the genus is considered to be monotypic. This species is widely distributed in wet lowland Neotropical forests and is common in large gaps, disturbed habitats, and older secondary forest. *Carludovica sulcata* (Fig. 1B) is a tall understory herb reaching 2.5 m in height. It is usually acaulescent and palm-like in form. The plants possess large underground rhizomes and readily reproduce vegetatively. This species is locally common in large gaps, disturbed habitats, and older secondary forest. A second species, *C. rotundifolia*, favors similar habitats but is more locally distributed at La Selva.

The Araceae is well represented in the understory flora of terrestrial herbs in Costa Rica with respect to both diversity of species present and the relative abundance of the species. Understory herbs comprise 25% of the 242 native species of Araceae in Costa Rica (Fig. 2). This group is represented by multiple species each of *Spathiphyllum*, *Dieffenbachia* and *Xanthosma*. Two genera which are more typically epiphytes or hemiepiphytes, *Philodendron* and *Athrurium* contribute terrestrial species as well. *Spathiphyllum fulvovirens* is the understory herb species with the highest frequency of occurrence in the primary forest understory at La Selva (Rundel et al., 2020). Understory aroids are less common in large gaps and along forest edges where solar irradiance is higher.

Many understory herbs in the Cyclanthaceae demonstrate important traits of efficient vegetative reproduction parallel to that well known to be present in herbaceous aroids. Most understory aroids have vegetative means of reproduction using rhizomes or stems with short internodes that creep over the soil and have shallow rhizomes although a few species with deep roots are notable. Examples of deeply rooted understory aroids at La Selva include *Philodendron grandipes* and *Anthurium ochranthum* (Croat, 1988), both relatively common and ecologically successful.

**Figure 5:**
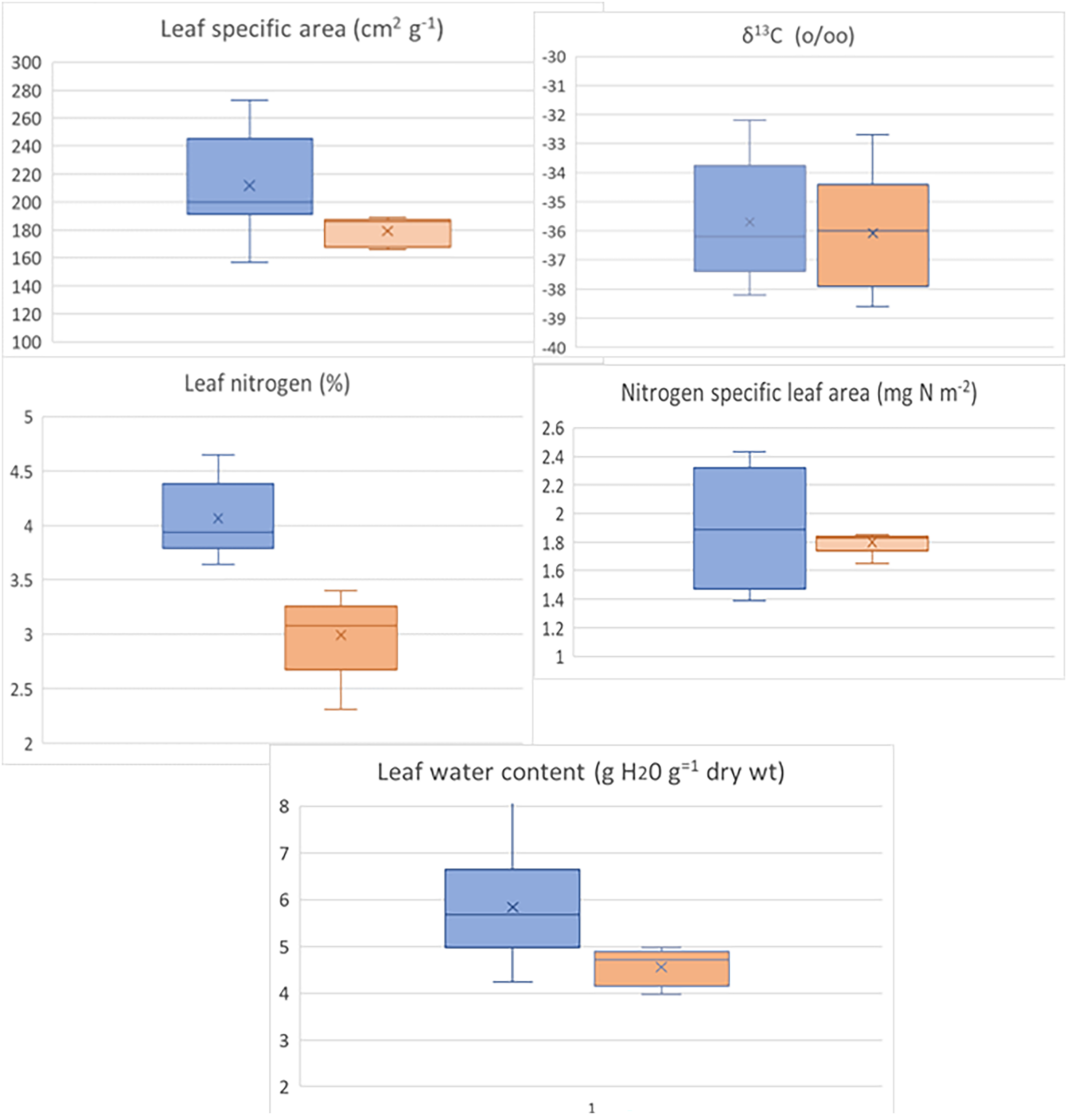
Trait comparison for terrestrial herbs between the Araceae (blue) and Cyclanthaceae (brick red). Data from Rundel et al. (2020). Only differences in leaf nitrogen content were significant with *p* < 0.05.

Mean values of functional leaf traits for understory herbs are similar between species of Cyclanthaceae and Araceae at La Selva. The values for leaf specific area, nitrogen specific leaf area, δ^13^C, and leaf water content do not differ significantly (Fig. 5). One notable exception is in the significantly higher concentration of nitrogen in herbaceous aroids. This level of leaf nitrogen is also significantly higher than that present in tropical Costaceae, Marantaceae, Heliconiaceae, and Zingiberaceae at La Selva (Rundel et al., 2020).

### Emergent aquatics, epilithic growth forms, and rheophytes

There are three small groups of unusual habitats that require specialized functional traits for survival and are present in a small number of species in the Cyclanthaceae and Araceae. These are emergent aquatics, epilithic growth forms, and rheophytes. The Costa Rican flora of Cyclanthaceae include rheophytes but no example of emergent aquatic aroids comparable to a small but significant group of aquatic species within the global Araceae. Some Costa Rican genera are exclusive to wetland habitats, as with *Montrichardia* and *Urospatha*, while some large genera have at least one species of rooted terrestrial whose natural habitat is standing water. An example of this can be seen with *Spathyphyllum friedrichthalii*, the only marsh species in its genus at La Selva. Overall, however, this aquatic habitat is relatively uncommon among Neotropical aroids.

Many species of the relatively large cyclanths genus *Dicranopygium* display an epilithic growth form that presents a special case of functional root traits that enable secure attachment to rocks. The root structure and development in these epilithic species allows the plants to firmly attach themselves to rocks by penetrating into the fine interstitial spaces within the rock (Mayo et al., 1997). These root traits are successful in establishing themselves on rock types that are sufficiently porous, with limestone being a prime example. Other species grow as appressed plants on tree trunks or on boulders. Porous limestone is particularly suitable for epilithic aroids because of its ability to catch debris and to provide many interfaces for adequate rooting sites but species restricted to limestone are rare. The presence of this trait in multiple species of the same genus suggests the possibility of highly evolved traits of the root rhizosphere. There are no identified epilithic species among the Araceae of Costa Rica although this growth specialization is present within Paleotropical members of the family (Croat, 1988).

The traits of epilithic growth are often associated with rheophytes that are restricted to growth on rocks in moving stream water. Rheophytes rooted in moving water present a specialized niche that combines characteristics of aquatic and epilithic habitats. These species cling to rocks on streambanks or within the stream itself (van Steenis, 1981, 1987). The presence of specialized rheophytes growing on rocks along the margins of fast-flowing streams can be found in in several species of *Dichranopygium* in Costa Rica, including *D. wedelii* at La Selva (Figs. 4C and 4D). This growth habit requires functional traits that allow the successful colonization of this highly specialized niche. Aroid rheophytes are widely present in the Paleotropics (Boyce & Wong, 2019). In contrast, rheophytic growth forms are uncommon in Neotropical aroids but do occur in a small number of species within the large genera *Anthurium* and *Spathiphyllum* (Croat, 1988).

### Epiphytes

**Figure 6:**
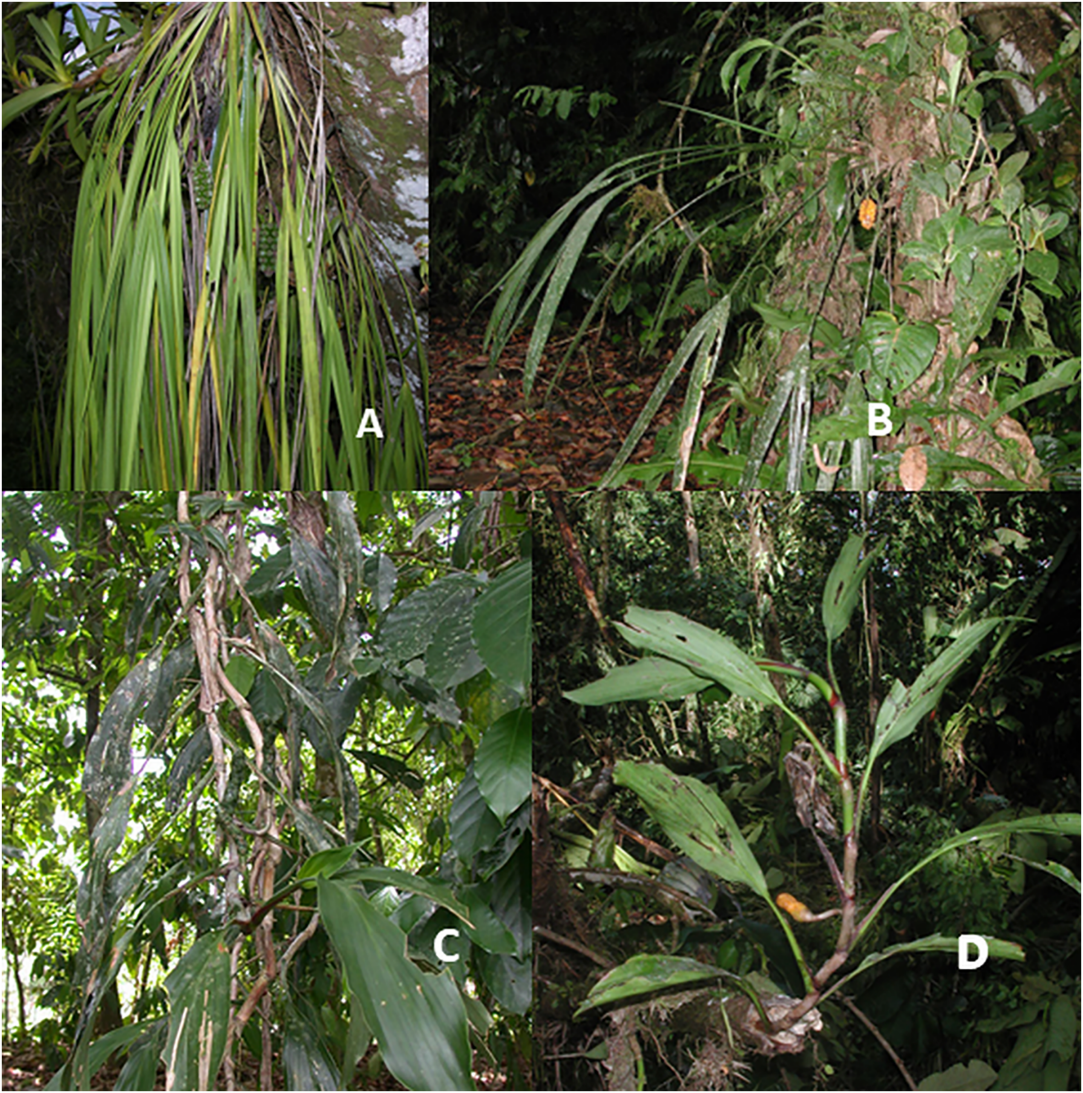
Epiphytic Cyclanthaceae. (A) *Chorigyne pendula*. (B) *Sphaeradenia acutitepala;* Photo by R. Aguilar (C) *Ludovia integrifolia* with liana growth form. (D) Young *Ludovia integrifolia* showing monopodial stem and entire leaves.

Epiphytes make up 28% (18 species) of the Cyclanthaceae in Costa Rica (Fig. 2). These epiphytes all fall into three genera which are represented in the flora of La Selva. *Corigyne pendula* at La Selva is one of seven species that occur only in Costa Rica and Panama (Fig. 6A). This acaulescent species colonizes large branches and is easy to recognize with its elongate gray-green leaves that hang vertically. It is a relatively common epiphyte in the La Selva forest. Although a true epiphyte, individual plants on larger fallen branches may survive for some time at ground level.

*Sphaeradenia* forms a relatively large genus of 53 described species characterized by short stems with long and narrow bifid leaves. The range of this genus extends from Nicaragua to northern South America. Some of these species can survive for some period of time on fallen branches but only rarely directly colonize terrestrial environments. *Sphaeradenia acutitepala* at La Selva is a shade-adapted epiphyte that typically grows more than 10 m above the ground, often as high as 20 m (Fig. 6B).

*Ludovia integrifolia* is an unusual epiphyte with a wide biogeographic distribution that extend from southern Mexico through Panama, where it is present on Barro Colorado Island (Croat, 1978), to northern South America. This species has smooth cylindrical stems up to 2–3 cm in diameter that display monopodial growth with short internodes between branches (Figs. 6C and 6D) These stems can extend for up to 10 m or more, leading some researchers to describe the species as a liana. In areas of treefalls the elongate stems may be seen dangling down into the lower canopy, giving them an appearance that can be mistaken for an aroid. The leaves are distinct from any other cyclanth at La Selva with the presence of simple blades with entire margins. *Ludovia lancifolia* from French Guiana is an epiphyte growing 20–25 m high in the canopy (Freiberg & Gottsberger, 2001).

Epiphytes form 28% (65 species) of the Costa Rican species of Araceae (Fig. 2). Much of the Neotropical richness in epiphytic Araceae lies within the genus *Anthurium*, and secondarily in a few species of *Philodendron*. There are several epiphytic lineages within *Anthurium* that develop short stems with associated a dense root mass, adapted to retain rainfall and stem flow of moisture and nutrients. Other species have evolved rosulate morphologies to catch and hold fallen debris in “baskets” formed by the leaves (Gerst et al., 2021). With this structure younger roots are able to grow into this debris, providing nutrients and an added capacity to hold water much as in many tank-forming bromeliads. Assessing the global Araceae, true epiphytes are abundant and diverse in wet Neotropical forests but notably uncommon in the Paleotropics. This difference in diversity of this growth form between the two floristic regions is largely the result of the presence of *Anthurium* and *Philodendron* in the Neotropics (Croat, 1988).

Several species of Araceae fit within a special category of species which begin their life as true epiphytes but develop extensive aerial roots which hang pendulously downward and eventually reach the ground and develop into full terrestrial root systems. There are a few examples of these so-called primary hemiepiphytes in *Philodendron* and *Anthurium* (Meyer & Zotz, 2004). The characteristics of a primary hemiepiphyte are better known in groups like strangler figs and Clusia (Putz & Holbrook, 1986; Holbrook & Putz, 1996; Williams-Linera & Lawton, 1995).

### Root-climbing hemiepiphytes

**Figure 7:**
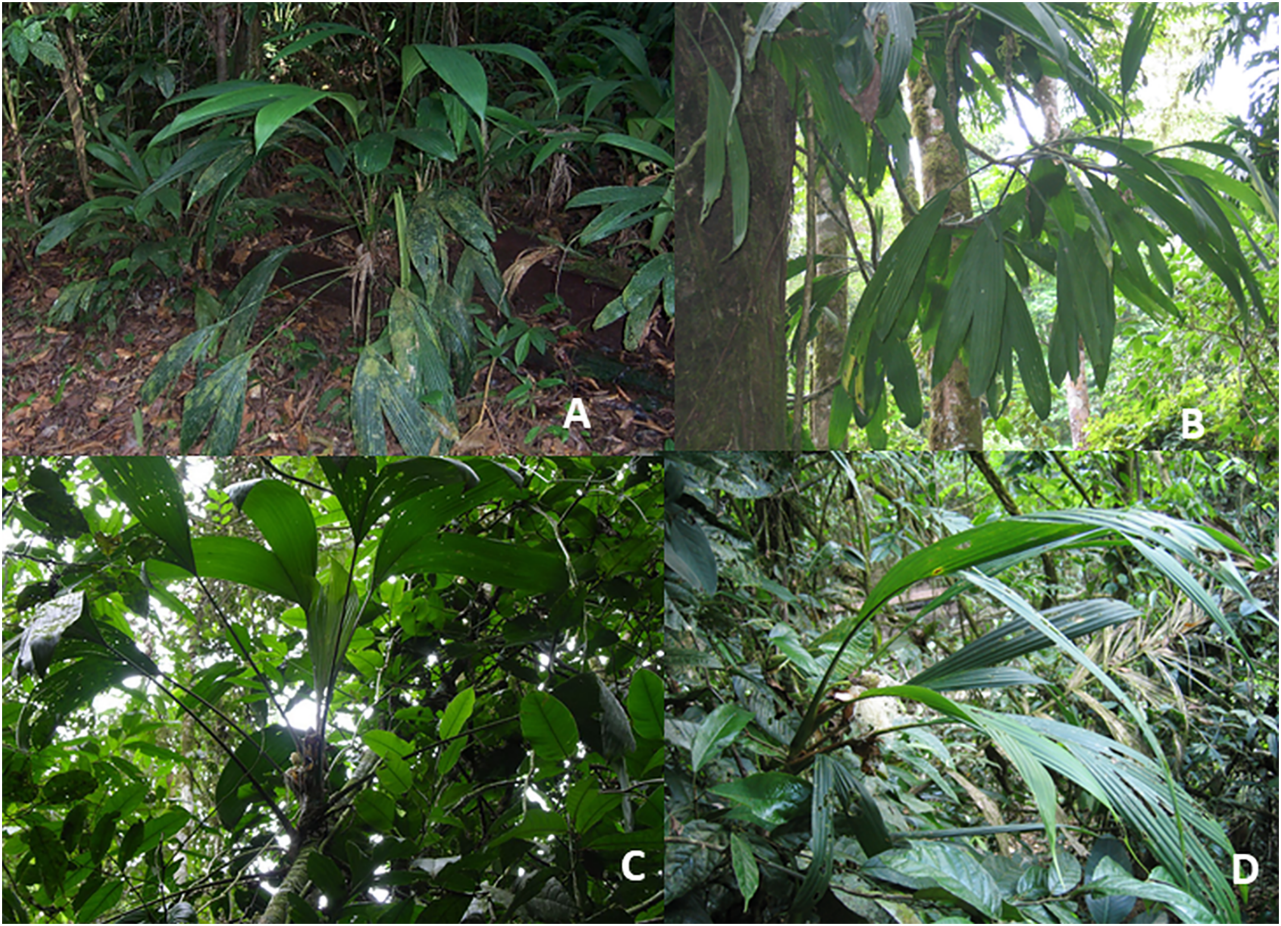
Root-climbing hemiepiphytes. (A) *Asplundia longitepala*. (B) *Asplundia multistaminata* (C) *Asplundia utilis*. (D) *Evodianthus funifer*.

The third functional group high in species richness in the Cyclanthaceae are the root-climbing hemiepiphytes, often referred to as secondary hemiepiphytes, which make up 21% (12 species) of the Costa Rican species (Fig. 2). A broad morphological diversity of root-climbing hemiepiphytes have evolved within *Asplundia* where this growth form is the most common functional trait of morphology with six of the eight species of *Asplundia* at La Selva. Five of these typically climb to intermediate trunk heights of 3–5 m, including *Asplundia longitepala* (Fig. 7A) and *Asplundia ferruginea*, both with a coarse unbranched stem. *Asplundia multistaminata* differs in having slender stems that branch repeatedly, forming tangled masses extending outward for 4 m or more in length laterally to produce a scandent form of growth (Fig. 7B; E. Riordan, 2022, unpublished data).

A few species extend higher into the canopy equal to many of the hemiepiphytic aroids. *Asplundia utilis* is one of the most common hemiepiphytes at La Selva in moist primary forest throughout the station up to 10 m (Fig. 7C). The root-climbing hemiepiphyte life history is also present in the monotypic *Evodianthus funifer*, characterized by long and slender stems which extend up to10 m into the canopy and branch freely (Fig. 7D). This species is frequent and widespread in moist primary forest at La Selva, especially on ridges and slopes (E. Riordan, 2022, unpublished data).

An impressive 45% (112 species) of the Araceae in Costa Rica are root-climbing hemiepiphytes (Fig. 2), a group with spectacular ecological success as indicated by their frequency of occurrence and large biomass (E. Riordan, 2022, unpublished data). The Neotropical aroid flora is particularly well known for a diversity of hemiepiphytes in the genera *Philodendron*, *Monstera*, *Syngonium*, *Rhodospatha* and *Heteropsis*. All of these genera are widespread and abundant in seasonally moist to very wet tropical forest in Costa Rica (Mayo et al., 1997).

The significance of the atrophy of the main stem early in the development in hemiepiphytesis a key trait that has not been well described. Experimental studies with hemiepiphytic *Monstera acuminata* showed that upper stem segments with more leaf area have wider vessels and that the production of new adventitious is necessary to prevent a lethal hydraulic constriction (López-Portillo et al., 2000).

One clue in the evolution of the root-climbing hemiepiphyte life history can be seen in our field observations of stem morphology in young developing hemiepiphytes. We found that four of the five cyclanths species we studies had 100% of their primary stem atrophied at an early stage of development (Table 2). All of these sampled species had well developed secondary roots that reestablish some degree of hydrologic connection to the soil. A slightly lower level of 85% loss of stem connection was observed in *Asplundia uncinata*, a species more typically terrestrial in growth with a full stem to soil connection. This life history was also found in *Dicranopygium umbrophilum*, an understory herb that may facultatively become a low climber and lose its direct hydraulic stem connection to the soil. For young hemiepiphytic Araceae we found a similar set of field observations that the main stem in was severed in as high a level as 85% in *Anthurium subsignatum* of our large sample size. The lowest level of severance was found in *Anthurium pentaphyllum* at 55%, with intermediate frequencies in *Philodendron alliodorum* (75%) and *Philodendron inaequilaterum* (67%).

## Discussion

The three functional growth forms of understory herb, epiphyte, and hemiepiphyte together characterize 98% of the Costa Rican species of Cyclanthaceae (Fig. 2). These groups of related functional traits nicely match those that have been described for the Araceae in Costa Rica (Croat, 1988; Croat & Ortiz, 2020). This is surprising given the distant evolutionary relationship between the two families. While terrestrial herbs and epiphytes are present in other tropical families, root-climbing hemiepiphytes exemplify a highly specialized growth form that is unusual within other Neotropical families. Root-climbing hemiepiphytes within the Araceae and Cyclanthaceae form a highly specialized group of taxa that have evolved the developmental, morphological, and ecophysiological traits necessary to achieve widespread ecological success.

Functional traits of leaf structure and morphology such as size, nitrogen content, and specific leaf area have been widely used across many ecosystems to predict community response to environmental change and the effects of changes in community composition on ecosystem processes (*e.g*., Lavorel & Garnier, 2002; Chapin et al., 2000; Díaz & Cabido, 2001). The development of a theory of leaf economics spectrum (LES) has shown that intercorrelated leaf traits related to construction costs per unit leaf area, nutrient concentrations, and rates of carbon fixation could link to complex ecological processes such as growth rate and productivity, specific leaf area and leaf nitrogen content (Reich, Walters & Ellsworth, 1997; Wright et al., 2005). It has been suggested that the use of community spectra of species traits to understand and predict community processes rather than a focus on individual species identity was a ‘Holy Grail’ in ecology (Funk et al., 2017). However, although broad tradeoffs among leaf structural and physiological traits have been demonstrated, we still do not have a comprehensive view of the fundamental constraints underlying the LES tradeoffs. Trait correlations are more complex and multi-dimensional.

The Cyclanthaceae with the parallel development of leaf ecophysiological traits and ecological limitations of plant morphology of root-climbing hemiepiphytes as well as terrestrial herbs and epiphytes provide a test in the application of functional trait values across distantly related plant families. Four morphological strategies of growth and development have been described for hemiepiphyte aroid root-climbers (Croat, 1988; Croat & Ortiz, 2020), and these can easily be applied to root-climbing hemiepiphytes in the Cyclanthaceae: (1) appressed trunk climbers, which grow flush against tree trunks; (2), scandent vines, which have a loose climbing habit (3) loosely branched scandent vines, which have a loose climbing habitat with low to moderate branching; and (4) highly branched scandent vines, which have a loose climbing habit and high degree of branching, often reaching high into the canopy (Croat, 1988). These growth strategies have significance in the canopy position of leaves and thus the photosynthetic responses to dynamic changes of irradiance in the canopy.

Most hemiepiphytes in the Araceae produce relatively long and slender internodes when they are young, but as the plants approach maturity there is a transition to shorter and thicker internodes. This pattern of repeated short internodes with slow vertical growth continues unchanged after sexual maturity is reached in most species. A variation of this growth pattern is present in scandent hemiepiphytes that do not make this transition to short internodes and continue perpetually in the production of long and slender internodes.

There is an important exception to this phenological pattern in many aroid genera that provides a flexibility of growth morphology that distinguishes them from the hemiepiphytes in the Cyclanthaceae as well as from tropical vines. This is developmental plasticity in stems and leaves growth termed heteroblasty (Zotz et al., 2020). Heteroblasty allows for repeated conversions back and forth from juvenile to adult growth with short, thick internodes to juvenile growth with long, slender stems (Ray, 1987a, 1987b; Mantovani, Pereira & Mantuano, 2017; Mantovani, Brito & Mantuano, 2018; Brito et al., 2022). Aroids with heteroblasty are very adept at repositioning themselves in relation to their environment and if necessary, repeatedly returning to the ground, seeking another source of support *and* climbing. At La Selva these include species of *Philodendron*, *Monstera*, *Rhodospatha*, *Heteropsis*, and *Syngonium*. Using this flexibility in phenological growth, they are able to modify their stems, petioles and leaves to adapt to their constantly changing needs (Ray, 1987a, 1987b; Croat, 1988). In addition, heteroblastic development also allows a plant the flexibility to cycle back and forth from juvenile to adult stem and leaf morphology. In this way, aroids are adept utilizing optimizing their placement of photosynthetic tissue in favorable canopy positions for optimal diurnal light exposure (Croat, 1988; Mantovani, Pereira & Mantuano, 2017; Mantovani, Brito & Mantuano, 2018; Zotz, Wilhelm & Becker, 2011). Heteroblasty is associated with the weedy growth of many aroid species. This trait coupled with rapid growth allows them to form dense tangled masses of stems along forest edges or disturbed sites.

### The evolution of root-climbing hemiepiphytes

There has been relatively little conjecture on the evolutionary development of root-climbing hemiepiphytes as a functional growth form with appropriate adaptive traits of morphology, ecophysiology and ecology. Root-climbing vines outside of the Araceae and Cyclanthaceae that might be classified as hemiepiphytes typically do not lose their stem connection to the soil with development.,The Marcgraviaceae is often cited as an example of hemiepiphytes outside of the Araceae and Cyclanthaceae. All *Marcgravia* species that we have observed at La Selva are rooted lianas or vines and not hemiepiphytes as we define this term.

The most puzzling aspect of the characterization of functional traits for root-climbing hemiepiphytes lies with the loss of the hydraulic connection of the main stem to the soil at a relatively early stage of growth. This condition seems at first thought to be counterintuitive as it would reduce hydraulic flow. With expanded growth of upper stem and upperleaves, however, the basal stem would provide a diminishing share of the water uptake by the plant. Our observations demonstrate that this severance of connection of the main stem commonly occurs at a young age before the plants are mature. We found that four of the five cyclanths species had 100% of their primary stem severed at this stage of development (Table 2). We also found in parallel observations that the main stem in young hemiepiphytic Araceae was severed in the majority of young plants but not at as high a frequency (55–97%) found in hemiepiphytic cyclanths (Table 2).

Other researchers have reported the common loss of main stem connection to the soil in diverse species of hemiepiphytic Araceae but at lower levels than we observed (Zotz et al., 2020; Bautista-Bello et al., 2021). These authors suggest that this loss of soil connection to the main stem is a hydraulic strategy related to the fact that almost all reported hemiepiphytes are monocotyledons that lack secondary growth. They suggest that the development of water uptake by adventitious roots may be the only option to allow sufficient flow to an increasingly larger shoot, making the lower portion of the stem expendable (López-Portillo et al., 2000). The dynamics of hydraulic structure of main stem roots and adventitious aerial roots in the Cyclanthaceae and Araceae remain poorly studied (but see Mantovani, Brito & Mantuano, 2018).

### Do hemiepiphytes exist as a distinct functional growth form?

A group of ecologists has recently questioned whether secondary hemiepiphytes should be categorized as a distinct growth form with adaptive traits distinct from other vine species (Zotz et al., 2021). The name *nomadic vine* has been proposed to replace the term *secondary hemiepiphyte* (Moffett, 2000) although this terminology has had mixed acceptance in the literature (see Zotz, 2013, 2020; Croat & Ortiz, 2020). The name nomadic vine seems a poor choice to us for several reasons. First, the proposed name adds the descriptor nomadic, which seems entirely inappropriate for characterizing the life history of these species. Nomads in human terms are classically wandering populations that lack a permanent home. The root-climbing hemiepiphytes are far from nomadic and utilize skototropism for directed growth toward dark areas which may represent large-tree diameters (Strong & Ray, 1975; Balcázar-Vargas et al., 2015). In this way they actively seek out permanent host trees.

There are functional traits that collectively distinguish hemiepiphytes from tropical vine that may utilize root-climbing adventitious roots. The trait of early loss of hydraulic connection of the main stem with the soil is typically present in climbing Araceae and Cyclanthaceae. Hydraulic connectivity to the soil is eventually replaced by adventitious roots that reestablish the soil connection (Mantovani, Brito & Mantuano, 2018). We found that this trait of dieback in the main stem was present in the great majority of the young stems of hemiepiphytic Cyclanthaceae and Araceae as they develop (Table 2).

*Marcgravia* species and other tropical forest vines with adventitious roots for climbing rarely survive after the main stem being severed above the soil line. In tropical root-climbing vines typically only the uppermost nodes typically bear leaves, and the main stem is intact and utilized for physical support as well hydraulic connections to the soil for water and nutrient uptake (Zona, 2020). The most active roots are associated with the leafy sections of the stem. In contrast, root-climbing hemiepiphytes often have leaves along their full length and roots are restricted to the nodes and scattered in occurrence. The stems lack a clear means of accumulating organic debris and associated nutrients compared to vines (Croat, 1988).

Additional evidence for the adaptive traits of the hemiepiphyte life form can be seen in examples of morphological traits that limit water loss and promote nutrient uptake after the primary stem rhizome is severed as the plants climb. These include compact root hairs, the development of more roots per node, or the presence of roots with velamen capable of extracting water directly from the air (Croat, 1981, 1988). In this manner, these are functionally similar to basins formed by leaf rosettes or clusters of stiff roots at the upper internodes that collect water and nutrients (Dressler, 1985; Zona & Christenhusz, 2015; Gerst et al., 2021).

## Conclusions

Although poorly studied, members of the Cyclanthaceae overall show a remarkable pairing of functional traits between with those that have been described for the Araceae. The proportional distribution of Cyclanthaceae among three functional groups (understory herbs, epiphytes, and root-climbing hemiepiphytes) is parallel with the distribution of species within the Araceae, suggesting that the two families share convergence in functional traits of morphology and life form evolution. The two families also show similarity in functional traits of pollination and seed dispersal (Gottsberger, 1991; Cockle, 2001; Silberbauer-Gottsberger et al., 2001; Henry & Jouard, 2007). There are multiple examples of parallel functional traits of canopy form and leaf traits among terrestrial herbs although leaf nitrogen concentrations terrestrial herbs of the Araceae are consistently higher than values for Cyclanthaceae and other understory herbs. The presence of root-climbing hemiepiphytes in the two families suggest a rare level of convergent evolution of this life history between Cyclanthaceae with *Asplundia* and *Evodianthus* and within the Araceae with diverse species of *Philodendron*, *Anthurium*, *Monstera*, *Rhodospatha*, *Heteropsis*, and *Syngonium* which are notable for their ecological success in terms of widespread occurrence, abundance, and biomass. One important functional trait that is present in many hemiepiphytic Araceae but apparently absent in hemiepiphytic Cyclanthaceae is heteroblasty in leaf and stem phenology. The evolution of heteroblasty may be significant in explaining the high species diversity and ecological success of hemiepiphytes in the Araceae compared with the Cyclanthaceae.

This remarkable parallel development of adaptive functional traits between the Cyclanthaceaand Araceae, despite the distant relationships of the families, suggests a convergence in evolutionary history in adapting to tropical forest ecosystems.

## Supplemental Information

10.7717/peerj.15557/supp-1 Supplemental Information 1 Leaf and canopy morphology raw data—morphological.

10.7717/peerj.15557/supp-2 Supplemental Information 2 Leaf ecophysiological trait raw data.

10.7717/peerj.15557/supp-3 Supplemental Information 3 Collection numbers for the voucher specimens for each study species archived in the herbarium of the La Selva Biological Station.

## Additional Information and Declarations

### Data Availability

The following information was supplied regarding data availability: The raw data for leaf traits is available in the Supplemental Files.
